# Comparative studies on osteogenic potential of micro- and nanofibre scaffolds prepared by electrospinning of poly(ε-caprolactone)

**DOI:** 10.1186/2194-0517-2-13

**Published:** 2013-11-14

**Authors:** Ting-Ting Li, Katrin Ebert, Jürgen Vogel, Thomas Groth

**Affiliations:** 1 Department Pharmaceutics and Biopharmaceutics, Biomedical Materials Group, Martin Luther University Halle-Wittenberg, Institute of Pharmacy, Heinrich-Damerow-Strasse 4, Halle (Saale), 06120 Germany; 2 GKSS Research Centre Geesthacht GmbH, Institute of Polymer Research, Max-Planck-Str.1, Geesthacht, 21502 Germany

**Keywords:** Poly(ε-caprolactone), Electrospinning, Microfibres, Nanofibres, Osteoblasts, Cell growth, Osteogenic activity

## Abstract

The biocompatibility and osteogenic potential of four fibrous scaffolds prepared by electrospinning of poly(ε-caprolactone) (PCL) was studied with MG-63 osteoblast cells. Two different kinds of scaffolds were obtained by adjustment of spinning conditions, which were characterized as nano- or microfibrous. In addition of one nanofibrous, scaffold was made more hydrophilic by blending PCL with Pluronics F 68. Scaffolds were characterized by scanning electron microscopy and water contact angle measurements. Morphology and growth of MG63 cells seeded on the different scaffolds were investigated by confocal laser scanning microscopy after vital staining with fluorescein diacetate and by colorimetric assays. It was found that scaffolds composed of microfibres stipulated better growth conditions for osteoblasts probably by providing a real three-dimensional culture substratum, while nanofibre scaffolds restricted cell growth predominantly to surface regions. Osteogenic activity of cells was determined by alkaline phosphatase (ALP) and o-cresolphthalein complexone assay. It was observed that osteogenic activity of cells cultured in microfibre scaffolds was significantly higher than in nanofibre scaffolds regarding ALP activity. Overall, one can conclude that nanofibre scaffold provides better conditions for initial attachment of cells but does not provide advantages in terms of scaffold colonization and support of osteogenic activity compared to scaffolds prepared from microfibres.

**Electronic supplementary material:**

The online version of this article (doi:10.1186/2194-0517-2-13) contains supplementary material, which is available to authorized users.

## Introduction

The traditional clinical methods of bone defect management, such as autografts and allografts of cancellous bone, can be limited by the large size of bone defects, poor viability of host environment and unpredictable graft resorption (Burg et al. 2000). Transplanting bone generated by autologous cells and tissue engineering could eliminate the problems of defect size, donor site scarcity, immune rejection and pathogen transfer. Cells, scaffolds and signals are the triad of tissue engineering, which is related to the main components of biologic tissues (Bell 2000). In order to grow into functional three-dimensional (3D) tissues or organs, osteoblasts progenitor cells require external signals including mechanical, structural and chemical cues. In actual bone tissue, the inhabitancy and the structural cue of cells are the extracellular matrix (ECM), which is composed of a composite meshwork of collagen fibres encased within a hard matrix of calcium phosphate (Burg et al. 2000). Hence, it would be desirable in tissue engineering of bone to apply biomaterials, which are organized as a three-dimensional scaffold-mimicking specific feature of the bone matrix, such as the fibrous elements of ECM (Guillame-Gentil et al. 2010).

Electrospinning is a versatile method for the production of fibres with diameters from micrometre to nanometre scale (Szentivanyi et al. 2011). It has gained serious interest for mimicking the structure of fibrillar extracellular matrix components and was proposed for applications in tissue engineering of bone as well (Yoshimatoa et al. 2003). Principally, electrospinning can be performed either with polymeric solutions or melts. In this process, the polymer solution or melt moves under the influence of a strong electric field between two electrodes. One of the electrodes is located in the spinning nozzle, while opposite to it, a grounded collector is located. As the polymer solution leaves the nozzle first, a hemispherical droplet is formed. If a critical strength of the electrical field is achieved, this form becomes conical, which is known as the Taylor cone. The polymer solution is ejected from this cone as the applied electric field is strong enough to overcome the surface tension of the fluid forming a jet that moves in a whipping mode to the grounded electrode. Due to the simultaneously occurring stretching of the jet and evaporation of the solvent, thin and solid fibres are deposited on the collecting electrode. The dimension and the morphology of the electrospun polymeric nanofibres can be influenced by a number of parameters such as composition of solution or blend, strength of the electric field, spinning distance, nozzle diameter, polymer concentration and the surface tension and electric conductivity of the polymer solution (Szentivanyi et al. 2011; Yoshimatoa et al. 2003; Gugutkov et al. 2013; Andiapann et al. 2013). Recent work has shown that electrospinning can be used to produce aligned fibres or combine fibres of different diameter in a sequential manner to achieve structures that guide cell adhesion, movement and differentiation (Gugutkov et al. 2013; Kim et al. 2010; Pham et al. 2006). This allows the generation of structures that resemble the composition of different tissues like the arterial wall, skin and other tissues (Gugutkov et al. 2013; Andiapann et al. 2013; Asran et al. 2010; Nottelet et al. 2009).

Poly(ε-caprolactone) (PCL) is an interesting material for tissue engineering application because of its non-toxic degradation products, controlled degradability and useful mechanical properties (Cruz et al. 2008). Electrospinning of biodegradable PCL and its application in tissue engineering has been reported recently (Pham et al. 2006; Nottelet et al. 2009; Kweon et al. 2003; Venugopal et al. 2006). It was also shown that interaction of cells with electrospun scaffolds could be significantly improved by modification of the surface chemistry and consequently, the hydrophobic/hydrophilic character of the polymer (Szentivanyi et al. 2011; Asran et al. 2010; Cruz et al. 2008). Indeed, as a weakness of nanofibre scaffolds, one can consider that usually a rather tight network is formed, which prevents penetration of cells. In addition, the mechanical properties of the obtained scaffold are rather weak. On the other hand, thicker fibres (in micrometre scale) provide better mechanical support but are less similar to ECM structure. Therefore, it was aimed here to study the influence of fibre dimensions, surface properties of fibres and the three-dimensional structure of scaffolds on adhesion, proliferation and differentiation of MG-63 human osteosarcoma cells. To achieve this goal, we varied the viscosity and conductivity of PCL solutions by additives like phosphate-buffered saline solution and change of voltage obtaining fibres of different diameter and scaffolds of lower density from microfibres and higher density from nanofibres. As additional parameter that could affect initial adhesion of cells, the wetting properties of the rather hydrophobic PCL fibres were improved by the addition of amphiphilic Pluronics F 68 to generate one type of hydrophilic nanofibrous scaffold. The results shall help to provide a better understanding how the scaffold architecture affects the behaviour of osteoblasts as a prerequisite for tissue engineering of bone.

## Methods

### Scaffold manufacturing conditions

PCL with a molecular weight of 80,000 was obtained from Sigma-Aldrich, Steinheim, Germany. Pluronics F-68 from Sigma, Steinheim, Germany was used as a non-ionic fluorescein diacetate (FDA) approved surfactant. All solvents were received from Merck, Darmstadt, Germany in p. a. quality. The PCL was dissolved in tetrahydrofurane (THF) and dimethylformamide (DMF) at room temperature until a homogeneous solution was obtained. The weight ratio of THF/DMF of 9:1 was equal for all solutions. Phosphate-buffered saline (PBS) tablets (Aldrich, Germany) were dissolved in 200 ml deionised water (0.01 M phosphate buffer, 0.0027 M KCl, 0.137 M NaCl). For one set of scaffolds, this PBS solution was added to the PCL solution to obtain a concentration of 1.44 wt.% PBS solution in the spinning solution to increase the conductivity of spinning solution. Prior to the spinning experiments, all solutions were heated for at least 15 min at 30°C under stirring and then filtrated through a 20-μm metal filter. Solution preparation and electrospinning were always performed within 1 day.

Electric conductivity of the spinning solutions was measured in a cell equipped with two oppositely deposited platinum electrodes. The cell was connected to a commercial conductivity meter LF 530 (WTW, Weilheim, Germany). About 2 to 3 ml of solution was used for conductivity measurements. Viscosities of the spinning solutions were measured with an Ubbelohde viscosimeter at 25°C (capillary IIIc for 16 wt.% PCL solutions, capillary IV for 18 wt.% PCL solutions). The viscosities given in Table 1 represent an average of three measurements.

**Table 1:** **Composition of solutions and spinning parameters**

Scaffold	Composition spinning solution					Distance [cm]	Voltage [kV]
	C_PCL_[%]	C_PluronicF68_[%]	C_PBS_[%]	Solvent	Viscosity [mm^2^/s]		
18%PCL	18	/	/	THF/DMF 9:1	1,598	25	35
18%PCL + Pluronic F68	18	1% of PCL	/	THF/DMF 9:1	1,585	27	25
16%PCL + PBS	16	/	1.44% of PCL	THF/DMF 9:1	1,202	25	25
16%PCL	16	/		THF/DMF 9:1	1,132	35	27

Electrospinning was performed with a set-up designed and constructed at GKSS Research Centre. The polymer solution was fed through a glassy capillary with an inner diameter of 0.2 mm. The spinning solution was in contact with a platinum electrode. The solution flow was adjusted with an infusion pump (Medipan Typ 610 BS, Medipan, Warsaw, Poland). The experiments were performed between 25 and 35 kV and a spinning distance between 25 and 35 cm to have another parameter to vary the fibre diameter. The electrospun fibres were collected on aluminium foils. The abbreviations used in this paper for the four PCL scaffolds electrospun from 18 wt.% PCL solution, 16 wt.% PCL solution, 18 wt.% PCL solution with an addition of Pluronic F 68, and 16 wt.% PCL/1.44 wt.% PBS are *18%*, *16%*, *18%* + *F 68*, and *16%* + *PBS*, respectively.

### Characterization of scaffolds

Static contact angle measurements were performed with a DSA 100 device from Kruess, Hamburg, Germany. A droplet of deionised water with a volume of 5 μl was deposited on the scaffolds on which the measurements were performed (sessile drop mode). The contact angles given in the paper are an average of 5 values measured within the first 5 s. The morphology of scaffolds was performed with a scanning electron microscope (Leo 1550 VP Gemini® field emission scanning electron microscope from Carl Zeiss Company, Jena, Germany). Samples were sputtered in a magnetron with a 2.5-nm-thick Au/Pd layer. The average distance of fibres was obtained by evaluation of scanning electron micrographs with Image J software measuring the largest distance of fibres on the surface region of scaffolds and naming this as mesh size as a parameter that should classify qualitatively the density of fibre network. The fibre diameter was also obtained from scanning electron micrographs by image analysis.

### Sample preparation and sterilization

The scaffolds on aluminium foil were cut into discs with a diameter of 14 mm. Replicates of each scaffold type were placed in 24-well culture plates. To avoid floating of scaffolds, glass rings were placed on the samples. Scaffolds were sterilized in 70% ethanol for 1 h and washed three times with distilled water. Scaffolds were kept in distilled water over night. The next day, distilled water was aspirated and substituted by culture medium. Before seeding, medium was removed and then replaced by the cell suspension.

### MG63 cell culture

MG63 cells were routinely cultured in 75- or 25-cm^2^ flasks at 37°C in a humidified incubator with 5% CO_2_. Cells were fed by standard Dulbecco's modified Eagle's medium (DMEM, Biochrom AG, Berlin, Germany) supplemented with 10% *v*/*v* fetal calf serum (FCS, PromoCell, Heidelberg, Germany), 1% Pen/Strep/Fungizone (PromoCell, Heidelberg, Germany) and 1% sodium pyruvate (Biochrom AG, Berlin, Germany). MG63 cells were seeded at a density of 10^4^ cells/ml in wells containing the sterilized scaffolds. Empty wells of tissue culture polystyrene plates were used as control. All plates were incubated at 37°C in a humidified incubator with 5% CO_2_. During the culture period, measurements were done on days 1, 3, 7, 10 and 14. For the measurement of calcium deposition MG-63 cells were cultured for 3 and 4 weeks. During these experiments osteogenic factors (L-ascorbic acid 2-phosphate (0.2 mM, Fluka, Steinheim, Germany), β-glycerophosphate (10 mM, Fluka, Steinheim, Germany) and dexamethasone (0.1 μM, Sigma, Steinheim, Germany) were added to the standard culture medium to support the osteogenic activity of MG-63 cells.

### Morphology and distribution of cells

Cell morphology was investigated by confocal laser scanning microscopy (CLSM) (LEICA DM IREZ TCS SP2 AOBS spectral confocal microscope, Leica Microsystems, Singapore) by staining MG-63 cells with FDA (Sigma, Steinheim, Germany). This allowed also studying the distribution of viable cells within the scaffolds. First, culture medium was aspirated and replaced by 1 ml fresh medium. Then, 5 μl FDA solutions (5 mg FDA/ml in acetone) was added to each well. After 5-min incubation at 37°C, the scaffold was transferred to a glass support slide, and the morphology and distribution of cells were evaluated by CLSM (excitation wavelength 485 nm, emission wavelength 520 nm).

### Cell viability and growth

Viability of cells was measured by QBlue assay (QBlue Cell Viability Assay Kits, BioChain, Newark, CA, USA). On the measuring day, the scaffolds were transferred to a new 24-well plate containing 500 μl of fresh medium. Fifty microlitres Qblue assay reagent was added to each well. Fresh medium without scaffold represented a blank value. After incubation for 2 h at 37°C, 100 μl medium was transferred from each well to a new black 96-well plate. Fluorescent intensity (excitation wavelength 544 nm, emission wavelength 590 nm) was measured with a fluorescence plate reader (BMG LABTECH, Fluostar OPTIMA, Offenburg, Germany).

Cell growth was measured by modification of the protocol of LDH Cytotoxicity Assay (WST-8 mix reagents, BioCat, Mountain View, CA, USA). On the measuring day, the scaffolds were transferred to a new 24-well plate. Cells were lysed with 0.5% TritonX-100 in distilled water for 30 min at 37°C. After that, the whole plate was centrifuged at 250 × *g* (1,300 rpm) for 10 min to remove cell debris. Ten microlitres cell lysis solutions was transferred from each well to a 96-well plate. Ten microlitres lysis solutions (0.5% Triton × 100) was used as blank reference. One hundred microlitres LDH reaction mixture was added into each well. The plate was incubated 30 min in room temperature without light. The absorbance was measured at 492 nm with a plate reader (BMG LABTECH, Fluostar OPTIMA, Offenburg, Germany).

### Measurement of osteogenic activity

Alkaline phosphatase (ALP) is a typical marker of early stage osteoblastic differentiation (Owen et al. 1990). Here, the quantification of ALP was determined by the hydrolysis of p-nitrophenylphosphate (pNPP, Roth, Karlsruhe, Germany) to p-nitrophenol (p-NP) at pH 10.2. p-NPP solution was prepared in bicarbonate buffer (NaHCO_3_) at pH 10.2 to obtain a concentration of 0.3 mg/ml. Fifty microlitres of residual cell lysis solutions (prepared for LDH assay) was transferred to a 96-well plate. Fifty microlitres lysis solution (0.5% TritonX-100 only) was used as blank reference. Then, the samples were incubated with 100 μl 0.3 mg/ml p-NPP solution for 1.5 h at 37°C. After incubation, ALP activity was determined by the absorbance at 405 nm using a plate reader (BMG LABTECH, Fluostar OPTIMA, Offenburg, Germany).

Deposited calcium phosphates form a purple-coloured complex with o-cresolphthalein complexone in an alkaline medium. 1.5 M AMP-Buffer (2-amino-2-methyl-3-propanol, Applichem) at pH 10.7 provides the proper alkaline medium for the colour reaction. After confluence (7-day culture), cells in one part of the 24-well plate were fed with inductor medium. Another half was cultured in DMEM without inductors. After 3 or 4 weeks, scaffolds were transferred into a new 24-well plate, 0.5 ml 0.6 M HCl was added into each well, including two wells without scaffolds as reference. After incubation over night at 37°C, 0.5 ml HCl suspension of each well was mixed with 0.5 solution of 0.16 mM o-cresolphthalein (Sigma, Steinheim, Germany) and 5 mM 8-Hydroxyquinoline-5-sulfate (HQS, VWR). HQS avoids an interference of the assay with by magnesium ions. The intensity of the colour was measured at 575 nm with a spectrophotometer SPECORD 200 (Analytik, Jena, Germany).

## Results

### Electrospinning and scaffold characterization

**Figure 1:**
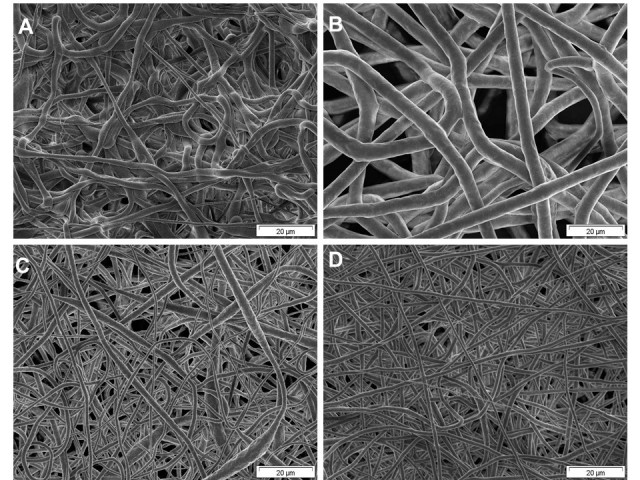
**Representative scanning electron micrographs of the different scaffolds. (A)** PCL scaffold electrospun from 18 wt.% PCL in THF/DMF 9:1 (voltage, 35 kV); spinning distance, 25 cm; nozzle, 0.2 mm. **(B)** PCL scaffold electrospun from 16 wt.% PCL in THF/DMF 9:1 (voltage, 27 kV); spinning distance, 35 cm; nozzle, 0.2 mm. **(C)** PCL scaffold electrospun from 18 wt.% PCL/0.18 wt.% Pluronics F 68 (voltage. 35 kV); spinning distance, 25 cm; nozzle, 0.2 mm. **(D)** PCL scaffold electrospun from 18 wt.% PCL/1.44 wt.% PBS (voltage, 25 kV); spinning distance, 25 cm; nozzle, 0.2 mm.

Representative scanning electron micrographs of the different scaffolds are shown in Figure 1. From scanning electron micrographs, the average fibre diameter as well as their density and distribution was estimated quantitatively and qualitatively as shown in Table 2 by image analysis with Image J. The fibre density was quantified as ‘mesh size’ indicating the average distance of fibres in horizontal direction. Scaffolds electrospun from 18 wt.% solutions showed rather thick fibres with diameters above 2 μm and only very few thin fibres with diameters of about 0.5 μm, which was related to the higher viscosity of the polymer solution. The mesh size of the fibre network was rather high with mean inter-fibre distances between 20 and 50 μm. Electrospun scaffolds obtained from a solution containing 18 wt.% PCL and Pluronic F 68 showed a rather non-uniform distribution of diameters with thin fibres of about 0.4 μm and a few very thick spindle-shaped fibres of about 6 μm in diameter. The addition of Pluronics F 68, which was done for making fibres more hydrophilic, had obviously also a huge effect on fibre diameter because neither viscosity nor spinning conditions were largely changed. The mesh sizes of the scaffolds prepared from 18%PCL spinning solutions with Pluronic F 68 was below 20 μm. Fibres obtained from the 16 wt.% solution were more uniform, no beads could be detected. However, with diameters above 5 μm, these fibres were much thicker than those obtained from the 18 wt.% solution. The mesh size of this scaffold was larger than 20 up to 100 μm. The reason for such rather unexpected finding may be the change of distance between capillary and collector from 25 cm for PCL 18% to 35 cm for PCL 16%. The addition of 1.44 wt.% PBS to a 16 wt.% PCL solution caused a significant increase in electric conductivity from 0.029 to 0.56 mS/m, which had a significant effect on fibre diameter. The resulting fibres of this solution showed a distribution of diameters between 0.6 and 2.8 μm. No beads were detected. The mesh sizes of the scaffolds obtained from the spinning solutions containing PBS were below 20 μm. According to the results of the diameter and mesh size measurements, meshes prepared from 16 and 18%PCL are called here microfibrous while 16% + PBS and 18% + F68 are denominated as nanofibrous scaffolds.

**Table 2:** **Characterization of scaffolds**

	Spinning solution			
Parameter	18%	18% + F68	16% + PBS	16%
Fibre diameter, mean ± SD, *n* = 20	2.7 ± 1.0 μm	939 ± 1,030 nm	937 ± 360 nm	5.0 ± 0.6 μm
Mesh size, *M*	20 < *M* < 50 μm	*M* < 20 μm	*M* < 20 μm	20 < *M* < 100 μm
Fibre density	++	+++	++++	+
Water contact angle [deg]	104.8 ± 4.1	46.6 ± 17.9	112.9 ± 4.9	101.1 ± 6.7

Table 2 displays also the wetting properties obtained by static water contact angle (WCA) measurements. WCA of the scaffolds electrospun from 16 wt.%, 16 wt.% + PBS and 18%PCL solutions did not differ significantly and were above 100°, which indicated the rather hydrophobic nature of all scaffolds spun from PCL. By contrast, the addition of Pluronics F 68 led a scaffold with much better wetting properties because the WCA of 18%PCL + F68 scaffold was about 50°. For all samples, a decrease in contact angle with time was observed (data not shown here), which is related to the porous character of the scaffold.

### Morphology and distribution of cells in/on the scaffold

**Figure 2:**
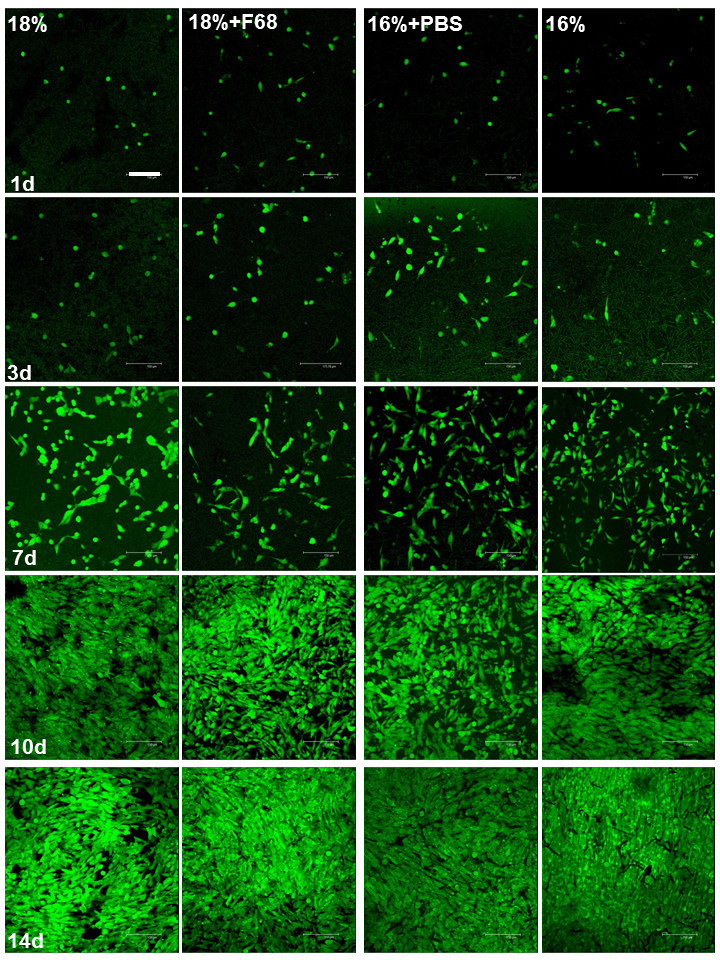
**Confocal micrographs of MG63 cells in scaffolds stained with FDA after different cultivated times.** The confocal images were taken along z-sections into the scaffolds and shown as 2D pictures composed by Leica Confocal Software. Scale bar, 150 μm.

**Figure 3:**
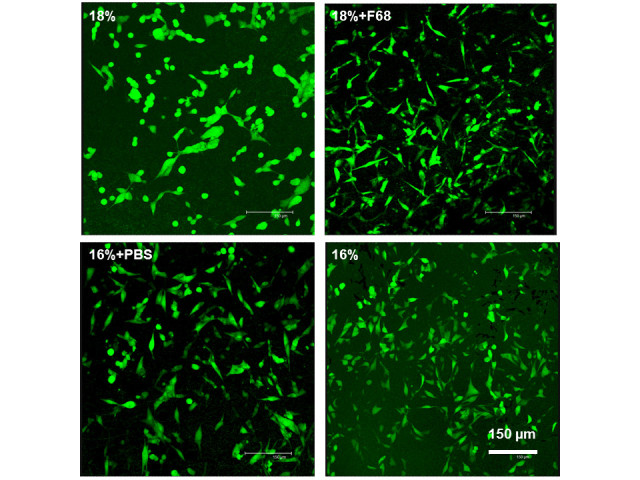
**CLSM pictures of MG63 cells in scaffolds stained with FDA after 7 days of culture.** Scale bar, 150 μm.

The morphology of viable MG 63 cells in the scaffold was characterized by confocal laser scanning microscopy (CLSM) after FDA staining. Cells were visualized along z-sections of confocal images in the scaffolds. In Figures 2 and 3, micrographs are shown as apparent two-dimensional (2D) images composed of all z-sections made, which was arranged by confocal microscopy software. This was done to visualize the total number of cells colonizing the scaffold within the range of visibility of confocal microscopy and presence of cells within the scaffold. Basically, all scaffolds supported MG 63 cell attachment and growth, which is demonstrated in Figure 2. It was observed that only a few adhering cells were found on all scaffolds with minor difference in their morphology after 24 h. An increase in cell numbers was observed after 3 days with small differences in morphology of cells between the scaffolds. Particularly MG-63 cells on the hydrophilic, nanofibrous 16% + F68 scaffolds developed a more elongated phenotype. These differences in morphology became more evident after 7 days of culture, which can be also seen in the higher magnification micrographs shown in Figure 3. MG 63 cells cultured on scaffolds with lower fibre diameters below the micrometre scale such as 16% + PBS and 18% + F68 promoted a more spread and elongated phenotype of cells, while cells growing on fibres with larger diameter like 16% and 18% were still round or growing in aggregates (see Figure 3 as well). After 10 days, cell density had increased greatly. Again, there was a notable difference between both major types of scaffolds. Those with lower fibre diameter (16% + PBS; 18% + F68) seemed to host less cells as indicated by the slightly lower density of cells. No major differences were observed after 14 days. Cell layers on all scaffolds appeared to be quite dense, though one should note that the images shown in Figures 2 and 3 represent a merge of different z-sections down to 70 μm inside the scaffold.

**Figure 4:**
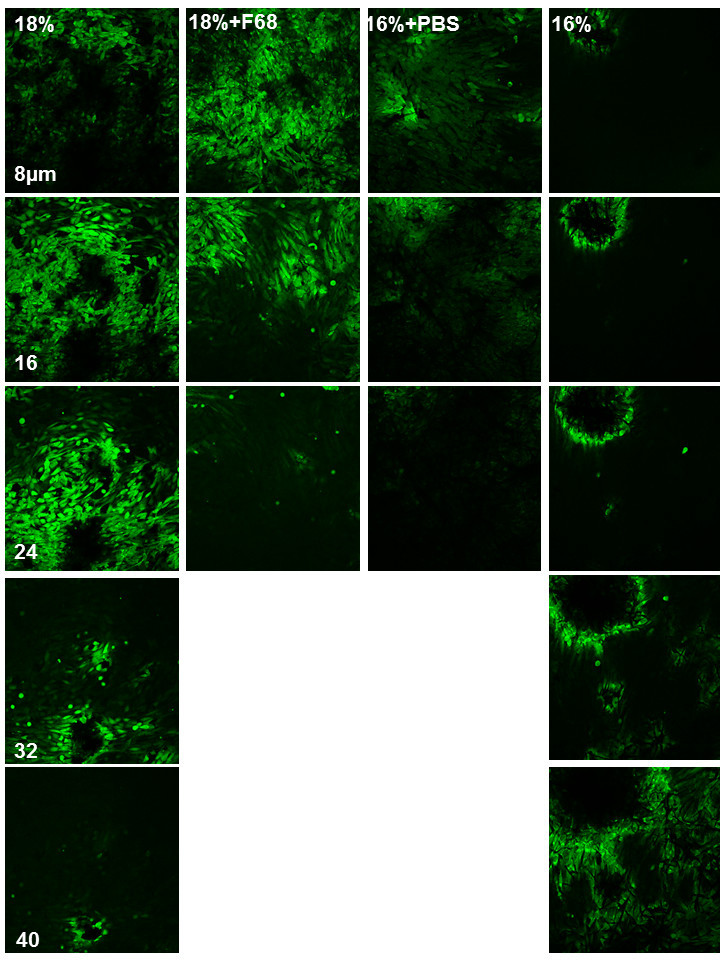
**Confocal micrographs of MG63 cells in scaffolds stained with FDA after 14 days of culture.** Five layers of confocal images were presented along z-sections from the surface to the inner part of scaffolds. Scale bar, 150 μm.

To show the distribution of cells within the scaffold, z-sections were made at different positions after 14 days of culture. Results are shown in Figure 4. Again, major differences were found between scaffolds composed of larger and smaller fibres. MG-63 cells cultured on nanofibre scaffolds (16% + PBS; 18% + F68 ) were growing predominantly in regions very close to the surface up to a depth of about 20 μm. No further viable cells were detected in lower regions of nanofibrous scaffolds. In opposite to this finding, cells cultured on 16% microfibre scaffold were growing predominantly in deeper regions down to 70 μm (data not completely shown in Figure 4), while MG 63 cells growing well in 18% microfibre scaffolds showed an apparent enrichment in central regions. In lower regions of 18% scaffolds only, some viable cells were found.

### Metabolic activity and growth of cells

**Figure 5:**
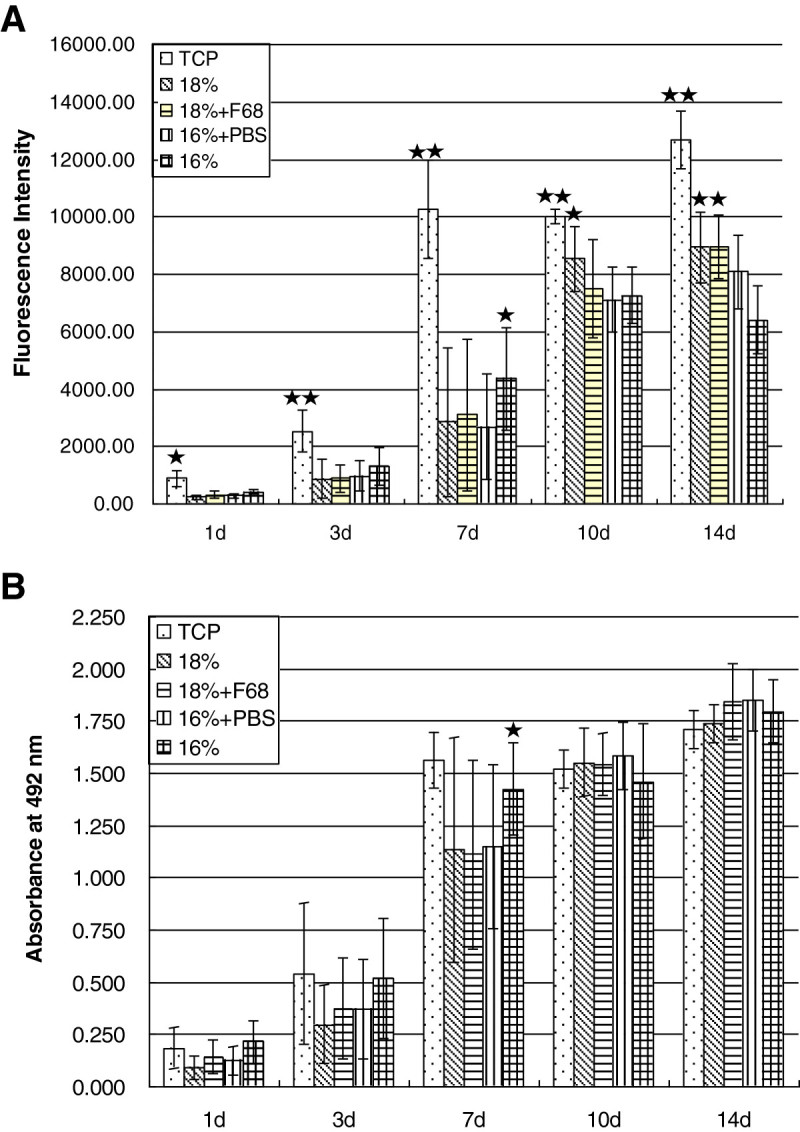
**Cell viability and cell growth. (A)** QBlue test; cells were cultured in standard medium in scaffolds and TCP as control fluorescence intensity was measured at excitation of 544 nm and emission of 590 nm. Values are mean ± SD, *n* = 8. The data were statistically analyzed with *t* test. **p < 0.001, there was a significant difference of cell viability between MG 63 cells seeded on TCP and scaffolds at each time point. ^*^p < 0.05, there was a significant difference of cell viability between MG 63 cells seeded on 16%PCL and the other three scaffold on day 7, and between 18%PCL and the other three scaffold on day 10, and between 18%PCL, 18%PCL + Pluronic and 16%PCL + PBS, 16%PCL on day 14. **(B)** Growth of MG63 cells cultured by standard medium in scaffolds and TCP as control. Absorbance was measured at 492 nm. Values are mean ± SD, *n* = 8. The data were statistically analyzed with a *t* test. ^*^p < 0.05, there was a significant difference of cell viability between MG 63 cells seeded on 16%PCL and the other three scaffold on day 7.

In addition to the morphological studies with CLSM, also quantitative data on metabolic activity and growth of cells were obtained. Figure 5A shows the results of experiments with QBlue to measure the metabolic activity of cells. Cells grown on tissue culture polystyrene (TCP) were considered as 2D control system here. It was observed that MG 63 cells cultured on tissue culture polystyrene (TCP) possessed always the highest metabolic activity during all days of measurement. It was also detected that metabolic activity increased during the culture period of 14 days with small and mostly non-significant differences between the different nanofibre scaffolds. This was also expected from the results of studies with vital cell staining and CLSM. There was one exception, namely 16%PCL where metabolic activity of cells was significantly lower than in all other samples. LDH assay was used here in a modified version (after induced lysis of cells) to estimate number of viable cells to quantify cell growth (see Figure 5B). It is interesting to note that the quantity of cells on TCP was only significantly higher up to 7 days and remained then comparable to cell quantities on/in the fibre scaffolds. By contrast, no such significant differences in cell quantities were observed with LDH proliferation assay among the different scaffolds. Only 16%PCL scaffold cells seemed to support cell grow more until day 7 measured by LDH assay in comparison to the other scaffolds, which was significant on day 7 only (*p* ≤ 0.05). It is also interesting to note that cell quantities in/on scaffolds measured LDH assay on days 7 and 14 were equal or slightly higher than on the 2D substratum TCP, while metabolic activity measured by QBlue assay was always highest on the control substratum.

### Osteogenic activity

**Figure 6:**
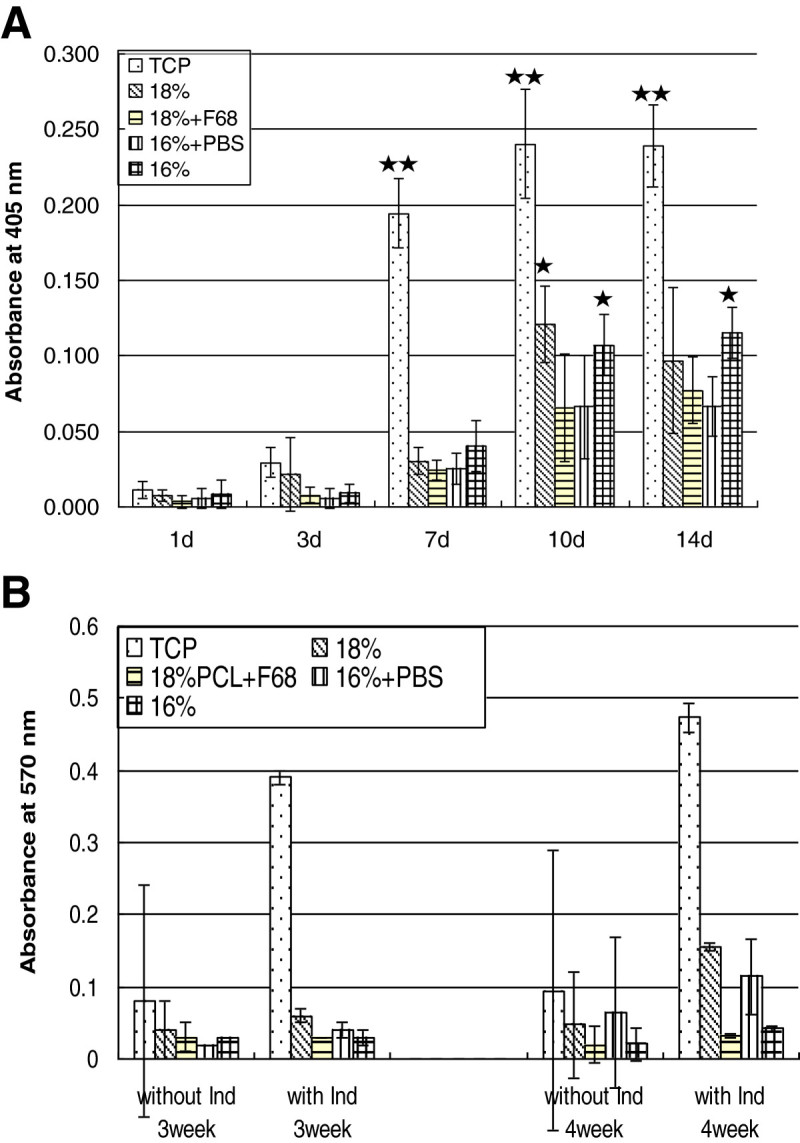
**The osteogenic activity of MG 63 cells cultured on the TCP and scaffolds. (A)** ALP activity of MG 63 cells cultured on the TCP and scaffolds after 1, 3, 7, 10 and 14 days. Absorbance was measured at 405 nm. Values are mean ± SD, *n* = 8. The data were statistically analyzed with a *t* test. **p < 0.001, there was a significant difference of ALP activity between MG 63 cells seeded on TCP and scaffolds at each time point. ^*^p < 0.05, there was a significant difference of ALP activity between MG63 cells seeded on microscaled scaffolds (16%PCL, 18%PCL) and nanoscaled scaffolds (18%PCL + Pluronic, 16%PCL + PBS) on day 10, and between 16%PCL and the other three scaffold on day 14. **(B)** MG63 cells seeded in scaffolds and on TCP were fed by standard medium and inductor medium after 1 week culture. The calcium deposition was measured by quantification of o-cresolphthalein complexone after 3 and 4 weeks. Optical density was measured at 570 nm. Values are means ± SD, *n* = 3.

The activity of alkaline phosphatase (ALP) was measured also after induced lysis of cells as an early marker of osteoblasts differentiation. Figure 6A shows ALP activity of MG 63 cells seeded in scaffolds and TCP over 14 days. The ALP activity of MG 63 cells on scaffolds increased until day 10 although an initial lag period was observed until day 3 for most of the scaffolds but not TCP and 18%. It was also found that ALP activity was always significantly greater in cells on TCP compared to the scaffolds. Beginning from day 7 on, microfibrous scaffolds 18% and 16% showed higher values than the nanofibrous scaffolds 18% + F68 and 16% + PBS. These differences were also significant (*p* ≤ 0.05).

The mineralization of calcium phosphate by MG 63 cells seeded on TCP and scaffolds was measured by quantification of o-cresolphthalein complexone. As shown in Figure 6B, after 3 and 4 weeks, the cells cultured with inductor medium always displayed more calcium deposition than the MG 63 cells in normal medium, which was also expected. It was also found that cells on TCP, cells treated by osteogenic medium showed almost five times more calcium deposition than the cells fed by standard medium both after 3 and 4 weeks culture. Under all conditions, mineralization of cells on TCP was much higher than in scaffolds. As the culture period prolonged, calcium deposition increased in all cases. Comparing the scaffolds, no remarkable differences were found after 3 weeks of culture. However, after 4 weeks, an obvious increase in calcium content was seen for cells cultured in 18%PCL and 16%PCL + PBS scaffolds.

## Discussion

In this study, the effect of fibre diameter and corresponding mesh sizes of electrospun PCL scaffolds on viability, growth and osteogenic activity of MG 63 osteoblast cell line were studied. By the adjustment of the polymer solution composition and spinning conditions, two PCL scaffolds were prepared that can be designated as microfibrous scaffolds (18%, 16%) with mesh sizes from 20 to 100 μm. By contrast, the two other scaffolds had a nanofibrous character (16% + PBS, 18% + F68) with mesh sizes below 20 μm (see also Table 2). As a general conclusion, one can state that nanofibrous scaffolds restricted the growth of cells predominantly to the surface, while microfibrous scaffolds allowed growth in deeper regions. The latter seemed also to enhance the osteogenic activity with regard to alkaline phosphatase activity, but this was also accompanied by a reduced metabolic activity of cells in one case (16% scaffold).

One observation during electrospinning of nanofibres from 18 wt.% PCL solutions was that the majority of fibres seemed to stick together at the points of contact. Most probably residuals of the lower volatile solvent DMF were still entrapped in the fibre at the time during deposition on the collector. This can usually be prevented by avoiding lower volatile solvents. However, because of the low dielectric constant of THF (*ε* = 7.52), the addition of DMF (*ε* = 38) was required to render the polymer solution electrically more conductive. Surprisingly, the scaffold made of 16%PCL solution showed significantly thicker fibres than those obtained from the higher concentrated solution, which was an unexpected finding. Normally, a decrease in polymer concentration usually causes also a decrease in fibre diameter (Szentivanyi et al. 2011; Yoshimatoa et al. 2003; Nottelet et al. 2009; Kweon et al. 2003). However, during the making of 16%PCL fibres, processing conditions were changed by reduction of voltage and increase of distance, which had a significant effect on fibre diameter increase. An improvement of uniformity of electrospun fibres can be achieved by the increase in electric conductivity of the spinning solution (Szentivanyi et al. 2011; Kweon et al. 2003). Therefore, usually organic salts or organic acids are added. However, in case of scaffolds applied in tissue engineering, biocompatibility and toxicity of additives have to be considered. For this reason, phosphate-buffered saline was used to increase the conductivity of 16 wt.% PCL solution. The resulting scaffolds showed a relatively uniform distribution of thinner fibres in the nanometer range. For rendering the hydrophobic PCL fibres, more hydrophilic Pluronic F 68 was added to the spinning solution as well. The resulting fibres were characterized by the lowest contact angle measured within this study (~47°), while all other scaffolds showed water contact angles above 100°.

Previous investigation showed that cell adhesion is generally improved on hydrophilic surfaces (Groth et al. 2010). However, in this study, 18% + F68 as the most hydrophilic scaffolds did not show a strikingly different response regarding cell attachment and proliferation. This confirms a previous report that wetting properties of 3D scaffolds do not strongly affect cell attachment and proliferation (Spasova et al. 2007). On the other hand, it was reported that the spreading of mouse fibroblasts was more supported on hydrophilic than on the hydrophobic electrospun surfaces (Bhattarai et al. 2006). Therefore, although hydrophilic scaffolds do not significantly promote cell proliferation, they might support cell spreading and increase viability of cells (Bacakova et al. 2004).

A noticeable difference was observed between the two microfibre scaffolds 18% and 16%. The viability and growth of cells were significantly higher in 16% scaffolds than in all other scaffolds before 10 days of culture. After that, there was no remarkably higher cell growth and the cell viability decreased in this scaffold after 14 days. In comparison to that, cell viability was significantly higher in 18% fibre networks after 10 days of culture. The characteristics of scaffolds (Table 2) show that 16% possessed the largest mesh size (sizes from 20 to 100 μm); followed by the mesh size of 18% nanofibre (sizes from 20 to 50 μm). Z-sections of confocal images revealed that the depth of cell growth inside scaffolds was consistent with the order of mesh size. Cells were growing deepest in 16% followed by 18% scaffolds. Indeed, MG63 cells in 18% were well-distributed in the 3D structure of the scaffold (Figure 5). The different behaviour of cells in the two micro-fibre scaffolds is probably due to the different pore structure of the scaffolds. Boudriot et al. suggested that the lack or at least reduced supply of oxygen and nutrients in the core of scaffolds diminishes metabolic activity of cells (Boudriot et al. 2006). In this study, the most porous scaffold 16% allowed adhesion of MG63 cells on the bottom area of the scaffolds, where they proliferated initially quite well. However, an increasing number of cells inside the scaffold increases the oxygen consumption and leads probably to hypoxic conditions after 10 days. By contrast, the well-distributed cells in 3D structure and the free space remained inside 18%PCL scaffolds provide better oxygen supply to the cells. This is also reflected by the reduced metabolic activity of cells in 16% scaffolds measured by QBlue assays, while cell quantities measured by the modified LDH assay were not different.

Previous studies suggested that nanofibre scaffolds with diameters like collagen fibres and 3D structures mimicking the natural extracelluar matrix promote cell adhesion, proliferation and differentiation (Gugutkov et al. 2013; Dzenis 2004). Also, Boudriot et al. showed with nanofibres from identical polymer solutions that the biocompatibility increased as the fibre diameter decreased (Boudriot et al. 2006). However, in this study, 18% + F68 and 16% + PBS scaffolds representing nanoscaled fibres did not showed significantly better biocompatibility than the microscaled fibre scaffolds 18% and 16%. The only advantage was more initial cell spreading during the early culture periods shown by CLSM (Figure 4). After 14 days of culture, the growth of most cells was still restricted to the surface region of the two nanofibre scaffolds (18% + F68, 16% + PBS). Obviously, the pore size of nanofibre scaffolds was not large enough to allow penetration of cells into the 3D structure. Therefore, the viability of cells could was affected by a reduced supply of oxygen. This result points to the important role of porosity of fibrous scaffolds as a major determinant of cell behaviour (Hutmacher 2000).

ALP is an early marker of osteogenic differentiation. ALP activity is present at high level in cells, which mineralized their matrix such as osteoblasts (Owen et al. 1990). The production of a mineralized matrix is considered as the final process of osteoblast phenotype in differentiation by quantification of calcium deposition (Owen et al. 1990; Bancroft et al. 2002). During osteoblast differentiation, L-ascorbic acid 2-phosphate, β-glycerophosphate and dexamethasone are used as osteogenic factors to effectively stimulate bone formation (Cheng et al. 1996). In this study, the cells treated by osteogenic inductor medium produced almost five times more calcium than the cells fed by standard medium both after 3 and 4 weeks culture. ALP expression follows immediately the down-regulation of cell proliferation. Later, the increasing expression of osteocalcein and osteopontin reflects the beginning of the mineralization step by deposition of the calciumphosphate hydroxyapatite (Owen et al. 1990). In this study, cells cultured on tissue culture polystyrene as control material (TCP) reduced their growth from day 7 on indicated by stagnant signal for LDH assay, while a dramatic increase of ALP activity was found on the same day. This shows that cells on TCP were confluent and started down-regulation of cell proliferation. On the four nanofibre scaffolds, ALP expression remained at a basal level while cells were still proliferating until day 7 (Figure 6A). That is the reason why the ALP activity decreased on day 7. This phenomenon indicates that the cell proliferation was lasting longer inside the scaffolds, which went along with delayed expression of ALP. It is also interesting to note that Koegler et al. modified PLGA scaffolds with different percentage of polyethylene oxide (PEO) - the hydrophilic block of the Pluronics copolymer. He found an increased ALP activity of MG63 cells on PLGA scaffolds coated with higher PEO concentrations (Koegler and Griffith 2004). However, in this study here, the most hydrophilic 18%PCL + Pluronic scaffold (1% Pluronic inside PCL polymer solution) did not express any stronger effect on MG63 cells differentiation. Moreover, it was observed during the differentiation studies that MG63 cells seeded in both microfibre scaffolds possessed a significantly higher ALP activity than in nanofibre scaffolds. However, calcium deposition after 4 weeks was higher only in one of the in microfibre 18%PCL scaffold, while also one of nanofibre 16%PCL + PBS scaffold showed also more calcium deposition. Here, the pore structure of electrospun scaffolds may play a key role in controlling the osteogenic response. Compared to 18%PCL scaffolds, the microfibre scaffold 16%PCL expressed high ALP activity but low calcium deposition, which could be related due to the lower metabolic activity of cells that grow on the bottom of the scaffold and suffer obviously from lack of oxygen. On the other hand, as the only scaffolds causing stronger aggregation of MG63 cells, high calcium deposition in 16%PCL + PBS scaffolds may demonstrate that nanofibre scaffolds can promote mineralization of matrix as well.

In comparison with TCP, all scaffolds showed much lower ALP expression and calcium deposition of MG63 cells. Van den Dolder et al. suggested that seeding at high cell density (8 × 10^5^ cells/ml) initially could further enhance calcification (Van der Dolder et al. 2002). High cell density is known to enhance cell-cell contacts and communication between osteoblasts and promote their differentiation (Cheng et al. 1998). Therefore, the lower ALP activity and calcium deposition on all scaffold materials might be due to the lower cell density during the culture compared to TCP. Previous work has also shown that even though biomaterial scaffolds support the growth and division of osteoblasts cells (MG63), they often alter their phenotypic expression, leading to a loss of their key characteristics, such as bone matrix formation (Zhang and Zhang 2004).

## Conclusions

In summary, all types of 3D scaffolds prepared in this study by electrospinning of poly(ε-caprolactone) were well biocompatible and supported proliferation of MG63 osteoblasts. The different hydrophilicity of 3D scaffolds did not affect cell adhesion and proliferation significantly as evident by the behaviour of cells on 18% + F68 scaffold. In general, MG63 cells seeded in microfibre scaffolds presented good viability and increased osteogenic activity regarding activity of ALP. Hence, it can be concluded that one important characteristic of scaffolds prepared by electrospinning is to promote osteoblasts function in 3D structures to provide an adequate porosity for cell growth inside the scaffold by arrangement of fibres to allow a homogenous distribution of cells. However, under conditions of oxygen transfer by diffusion only, the metabolic activity of cells can be hampered by insufficient oxygen supply due to the growth inside the scaffolds. Compared to the cells in microfibre scaffolds, the growth of cells seeded on nanofibre scaffolds expressed better initial cell spreading, which may affect growth and differentiation of osteoblasts in a positive manner. Although the ALP expression of osteoblasts in nanofibre scaffolds was remarkably lower, high calcium deposition was observed in one of them (16%PCL + PBS) scaffolds after long-term culture. This indicates at least in line with previous studies that nanofibre scaffolds are also quite useful for promoting osteogenic activity (Kweon et al. 2003; Zhang and Zhang 2004). The obvious disadvantage of nanofibre scaffolds to block entry of cells due to too close deposition of fibres can be overcome by a combination of nano- and microfibres to a suitable scaffold architecture with sufficient mechanical strength that allows penetration of cells into the structure but provides ale specific topographical stimuli to cells due to interaction with nanofibres as shown by some recent work of different groups (Kim et al. 2010; Pham et al. 2006; Tuzlakoglu et al. 2005).

## Authors’ original submitted files for images

Below are the links to the authors’ original submitted files for images. Authors’ original file for figure 1 Authors’ original file for figure 2 Authors’ original file for figure 3 Authors’ original file for figure 4 Authors’ original file for figure 5 Authors’ original file for figure 6 Authors’ original file for figure 7

## References

[CR1] Andiapann M, Sundaramoorthy S, Panda N, Meiyazhaban G, Winfred SB, Venkatamaran G, Krishna P (2013). Electrospun eri silk fibroin scaffold coated with hydroxyapatite for bone tissue engineering applications. Progress Biomater.

[CR2] Asran AS, Razghandi KH, Aggarwal N, Michler GH, Groth T (2010). Nanofibers from blends of polyvinyl alcohol and polyhydroxybutyrate as potential scaffold material for tissue engineering of skin. Biomacromolecules.

[CR3] Bacakova L, Filova E, Rypacek F, Svorcik V, Stary Y (2004). Cell adhesion on artificial materials for tissue engineering. Physiol Res.

[CR4] Bancroft GN, Sikavitsas VI, van der Dolder J, Sheffield TL, Ambrose CG, Jansen JA, Mikos A (2002). Fluid flow increases mineralized matrix deposition in 3 D perfusion culture of marrow stromal osteoblasts in a dose dependent manner. Proc Natl Acad Sci U S A.

[CR5] Bell E, Vacanti JP (2000). Tissue Engineering in perspective. Principles of tissue engineering.

[CR6] Bhattarai SR, Bhattarai N, Viswanathamurthi P, Yi HK, Hwang PH, Kim HY (2006). Hydrophilic nanofibrous structure of polylactide; fabrication and cell affinity. J Biomed Mat Res.

[CR7] Boudriot U, Dersch R, Greiner A, Wendorff JH (2006). Electrospinning approaches toward scaffold engineering - a brief overview. Artif Organs.

[CR8] Burg KJL, Porter S, Kellam JF (2000). Biomaterial development for bone tissue engineering. Biomaterials.

[CR9] Cheng SL, Zhang SF, Avioli LV (1996). Expression of bone matrix proteins during dexamethasone-induced mineralization of human bone marrow stromal cells. J Cell Biochem.

[CR10] Cheng SL, Lecanda F, Davidson MK, Warlow PM, Zhang SF, Zhang L, Suzuki S, St. John T, Civitelli R (1998). Human osteoblasts express a repertoire of cadherins, which are crucial from BMP-2 induced osteogenic differentiation. J Bone Miner Res.

[CR11] Cruz DMG, Coutinho DF, Martinez EC, Ribelles JLG, Sanchez MS (2008). Blending polysaccharides with biodegradable polymers II: structure and biological response of chitosan/polycaprolactone blends. J Biomed Mat Res B: App Biomater.

[CR12] Dzenis Y (2004). Spinning continuous fibers for nanotechnology. Science.

[CR13] Groth T, Liu Z-M, Niepel M, Peschel D, Kirchhof K, Altankov G, Faucheux N, Shastri VP, Altankov G, Lendlein A (2010). Chemical and physical modifications of biomaterials surfaces to control adhesion of cells. Advances in regenerative medicine: role of nanotechnology and engineering principles.

[CR14] Gugutkov D, Gustavsson J, Ginebra MP, Altankov G (2013). Fibrinogen nanofibers for guiding endothelial cell behavior. Biomater Sci.

[CR15] Guillame-Gentil O, Semenov O, Roca A, Groth T, Zahn R, Vörös J, Zenobi-Wong M (2010). Engineering the extracellular environment: strategies for building 2D and 3D cellular structures. Adv Materials.

[CR16] Hutmacher DW (2000). Scaffolds in tissue engineering of bone and cartilage. Biomaterials.

[CR17] Kim SJ, Jang DH, Park WH, Min B-M (2010). Fabrication and characterization of 3-dimensional nanofibre/microfiber scaffolds. Polymer.

[CR18] Koegler WS, Griffith LG (2004). Osteoblast response to PLGA tissue engineering scaffolds with PEO modified surface chemistries and demonstration of a patterned cell response. Biomaterials.

[CR19] Kweon H, Yoo MK, Park IK, Kim TH, Lee HC, Lee HS, Oh JS, Akaike T, Cho CS (2003). A novel degradable polycaprolactone networks for tissue engineering. Biomaterials.

[CR20] Nottelet B, Pektok E, Mandracchia D, Tille J-C, Walpoth B, Gurny R, Möller M (2009). Factorial design optimization and in vivo feasibility of poly(e-caprolactone)-micro- and -nanofiber based small diameter vascular grafts. J Biomed Mat Res.

[CR21] Owen TA, Aronow M, Shalhoub V, Barone LM, Wilming L, Tassinari MS, Kennedy MB, Pockwinse S, Lian JB, Stein GS (1990). Progressive development of the rat osteoblast phenotype in vitro: reciprocal relationships in expression of genes associated with osteoblast proliferation and differentiation during formation of the bone extracellular matrix. J Cell Physiol.

[CR22] Pham QP, Sharma U, Mikos AG (2006). Electrospun poly(ε-caprolactone) microfiber and multilayer nanofibre/microfiber scaffolds: Characterization of scaffolds and measurements of cellular infiltration. Biomacromolecules.

[CR23] Spasova M, Stoilova O, Manolova N, Raskov I (2007). Preparation of PLLA/PEG nanofibers by electrospinning and potential applications. J Bioact Compat Polymers.

[CR24] Szentivanyi AL, Zernetsch H, Menzel H, Glasmacher B (2011). A review of developments in electrospinning technology: new opportunities for the design of artificial tissue structures. Int J Artif Organs.

[CR25] Tuzlakoglu K, Bolgen N, Salgado AJ, Gomes ME, Piskin E, Reis RL (2005). Nano and microfiber combined scaffolds: a new architecture for bone tissue engineering. J Mat Sci Mat Med.

[CR26] Van der Dolder J, Vehof JWM, Soauwen PHM, Jansen JA (2002). Bone formation by rat bone marrow cells cultured on titanium fiber mesh: effect of culture time. J Biomed Mat Res.

[CR27] Venugopal JR, Zhang Y, Ramakrishna S (2006). In vitro culture of human dermal fibroblasts on electrospun polycaprolactone collagen nanofibrous membrane. Artif Organs.

[CR28] Yoshimatoa H, Shina YM, Teraia H, Vacanti JP (2003). A biodegradable nanofiber scaffold by electrospinning and its potential for bone tissue engineering. Biomaterials.

[CR29] Zhang Y, Zhang M (2004). Cell growth and function on calcium reinforced chitosan scaffolds. J Mat Sci Mat Med.

