# Supplementary figures and images for: Comparative studies on osteogenic potential of micro- and nanofibre scaffolds prepared by electrospinning of poly(ε-caprolactone)
Source: Prog Biomater. 2013 Nov 14;2:13. doi: 10.1186/2194-0517-2-13 (PMC5151106; doi:10.1186/2194-0517-2-13)

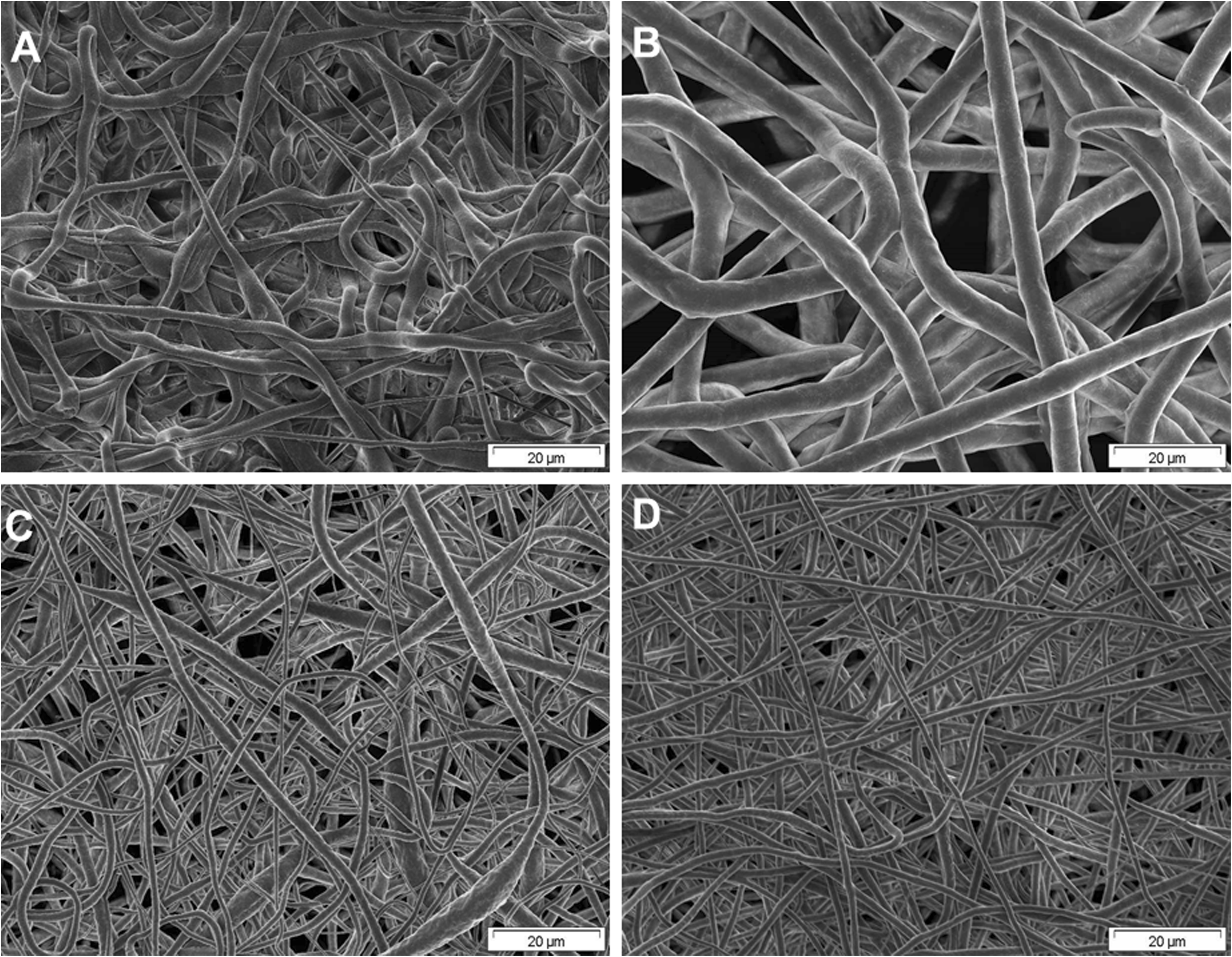

Supplement: Supplementary file 1 — Authors’ original file for figure 1 [file 40204_2013_17_MOESM1_ESM.tiff]

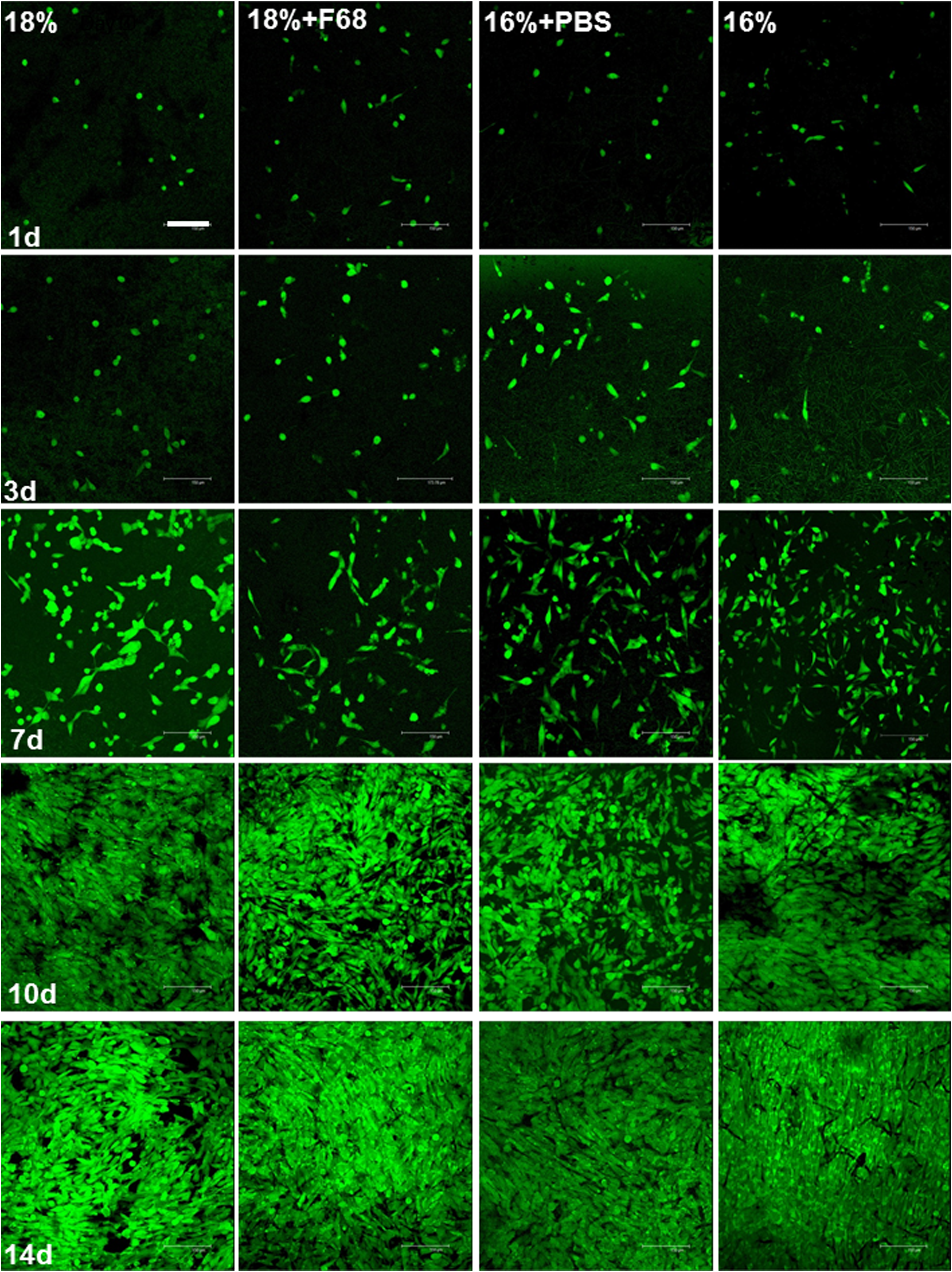

Supplement: Supplementary file 2 — Authors’ original file for figure 2 [file 40204_2013_17_MOESM2_ESM.tiff]

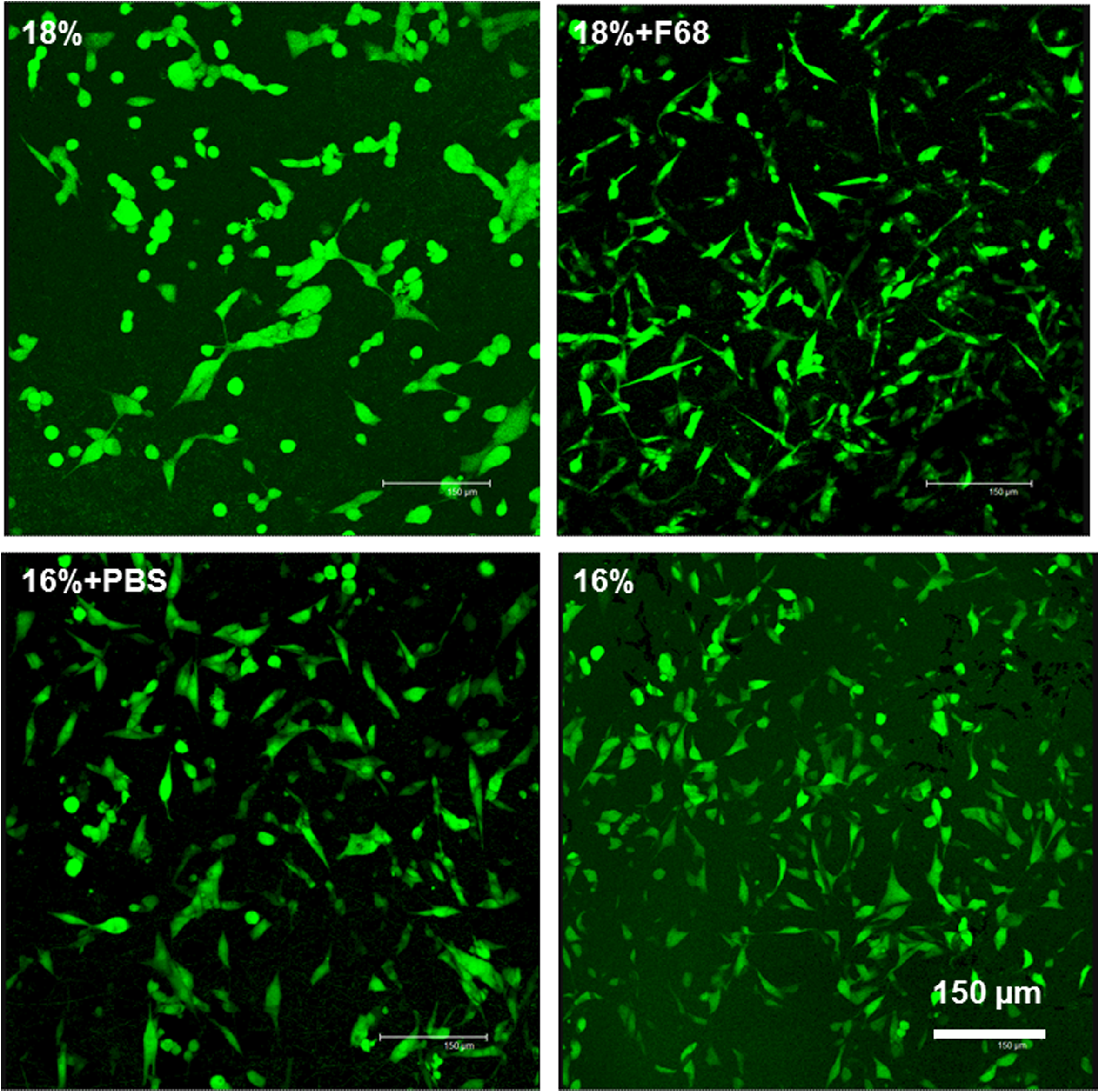

Supplement: Supplementary file 3 — Authors’ original file for figure 3 [file 40204_2013_17_MOESM3_ESM.tiff]

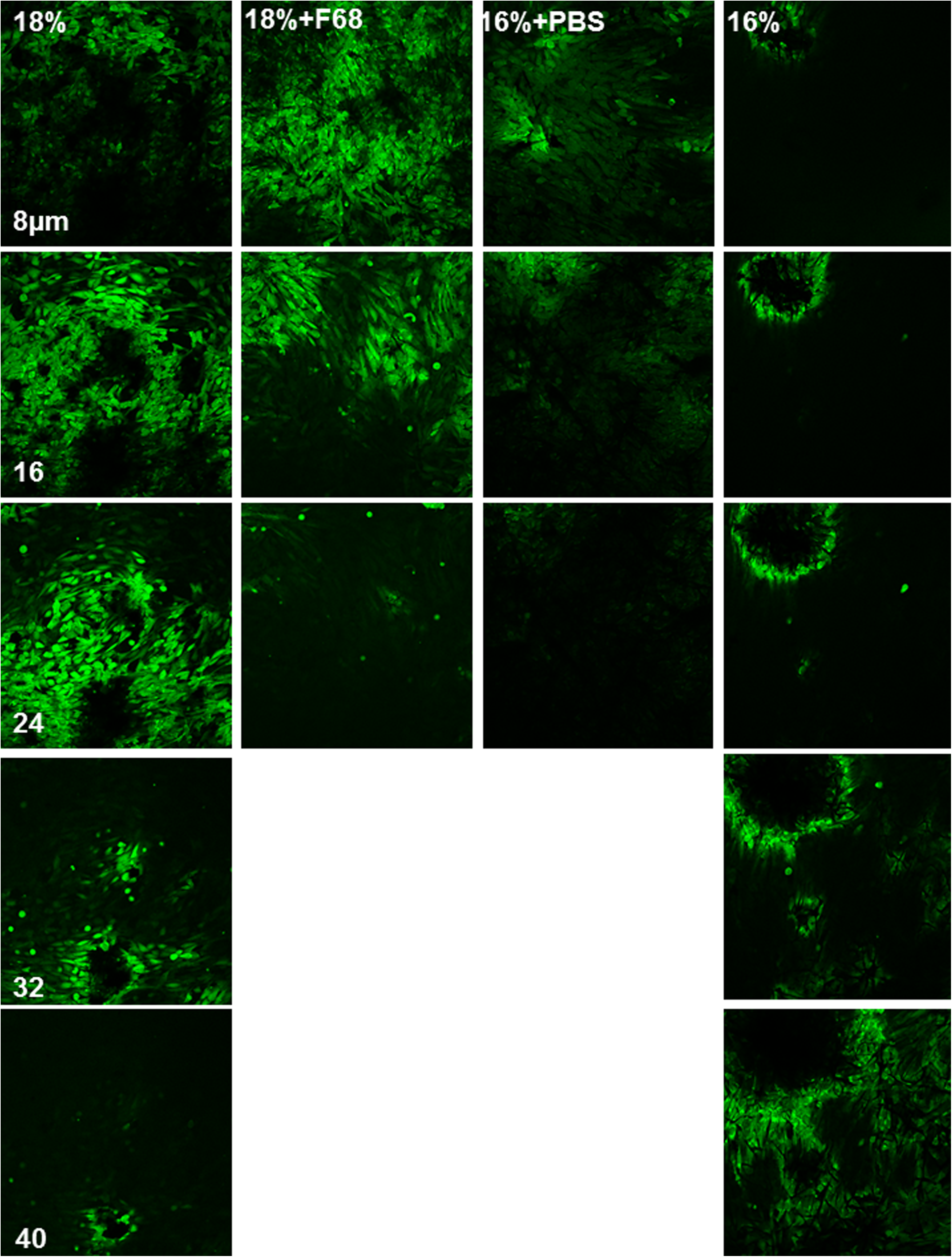

Supplement: Supplementary file 4 — Authors’ original file for figure 4 [file 40204_2013_17_MOESM4_ESM.tiff]

**A**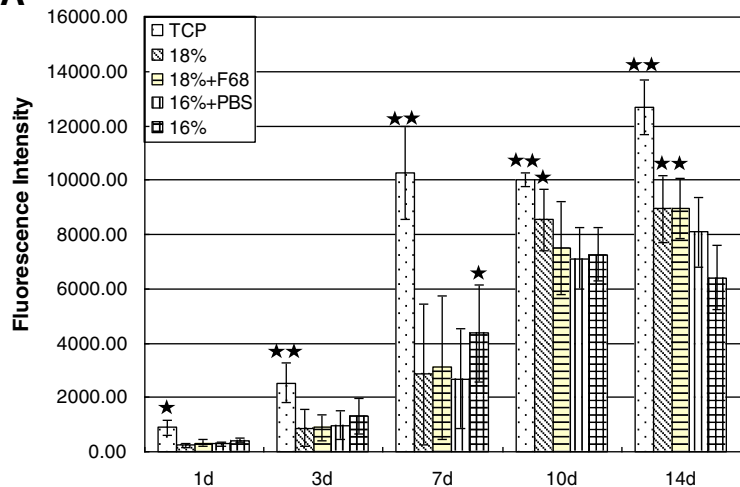**B**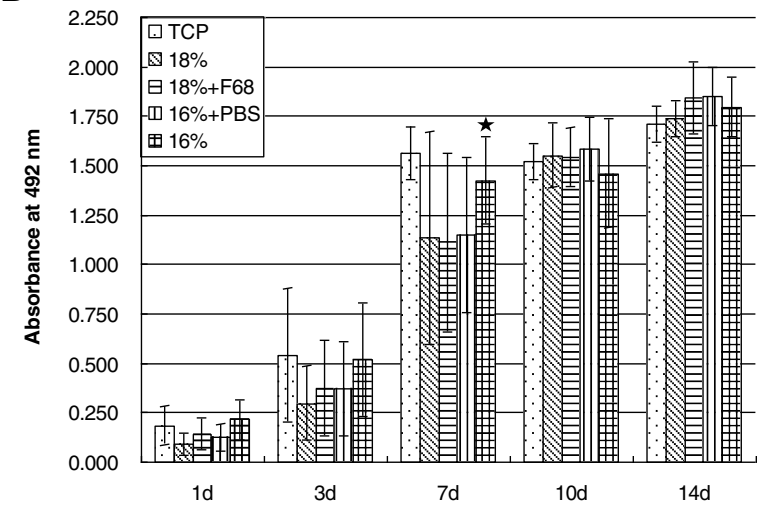

Supplement: Supplementary file 5 — Authors’ original file for figure 5 [file 40204_2013_17_MOESM5_ESM.pdf]

**A**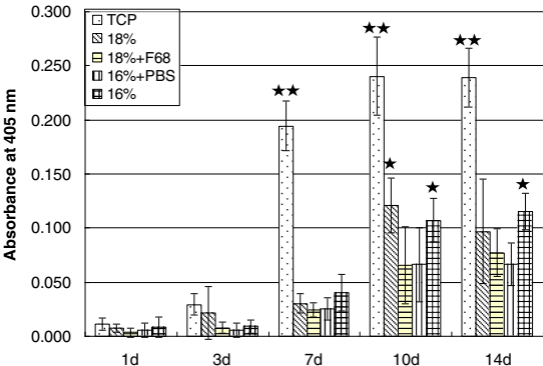**B**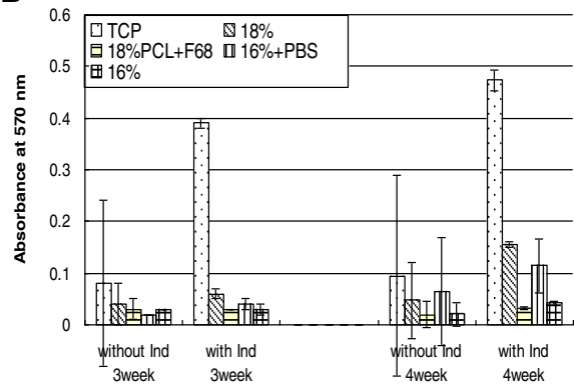

Supplement: Supplementary file 6 — Authors’ original file for figure 6 [file 40204_2013_17_MOESM6_ESM.pdf]
