# Supplementary material for: Comparative studies on osteogenic potential of micro- and nanofibre scaffolds prepared by electrospinning of poly(ε-caprolactone)
Source: Prog Biomater. 2013 Nov 14;2:13. doi: 10.1186/2194-0517-2-13 (PMC5151106; doi:10.1186/2194-0517-2-13)
Supplement: Supplementary file 7 — Authors’ original file for figure 7 [file 40204_2013_17_MOESM7_ESM.docx]

**Figure 7**

#

**A**

**B**
